# Effects of roxadustat on thyroid hormone levels and blood lipid metabolism in patients undergoing hemodialysis: a retrospective study

**DOI:** 10.7150/ijms.97599

**Published:** 2024-07-09

**Authors:** Nan Li, Wenxia Cui, Dinghuang Mu, Xiaoting Shi, Lei Gao, Sixiu Liu, Hengjin Wang, Chunming Jiang, Yun Hu

**Affiliations:** 1 Department of Nephrology, Nanjing Drum Tower Hospital, Drum Tower Clinical College of Nanjing Medical University, Nanjing, China.; 2 Department of Geriatrics, Nanjing Drum Tower Hospital, Drum Tower Clinical College of Nanjing Medical University, Nanjing, China.; 3 Department of Geriatrics, Nanjing Drum Tower Hospital Clinical College of Nanjing University of Chinese Medicine, Nanjing, China.; 4 Department of Nephrology, Nanjing Drum Tower Hospital, Affiliated Hospital of Medical School, Nanjing University, Nanjing, China.; 5 Department of Geriatrics, Nanjing Drum Tower Hospital, Affiliated Hospital of Medical School, Nanjing University, Nanjing, China.; 6 Department of Chemistry, State Key Laboratory of Analytical Chemistry for Life Science, Nanjing University, Nanjing, China.

**Keywords:** roxadustat, hemodialysis, thyroid hormones, blood lipid, anemia

## Abstract

**Background:** Roxadustat is commonly used to treat renal anemia. However, the potential effects of roxadustat on metabolism and organs other than the kidneys have recently attracted increased attention.

**Objective:** This study aimed to examine the regulatory effects of roxadustat on thyroid hormones and blood lipid metabolism in patients with end-stage kidney disease (ESKD) undergoing hemodialysis.

**Methods:** Eighty ESKD patients on hemodialysis and taking roxadustat were enrolled. Hemoglobin, thyroid hormones (TSH, FT3, FT4), and blood lipid profiles (TC, LDL-C, TG, HDL-C) were assessed before and after treatment. Changes in these parameters were compared, and relevant causative factors were analyzed.

**Results:** Roxadustat significantly increased Hb, lowered TSH, FT4, TC, and LDL-C levels (all P<0.001). Patients were categorized into three groups based on post-treatment TSH inhibition percentage: Q1(≥70%), Q2(30%-70%), Q3(≤30%). Pre-treatment TSH decreased with reduced TSH inhibition (P<0.05). Post-treatment, TC, LDL-C, TSH, FT3, and FT4 increased with reduced TSH inhibition (all P<0.05).TC and LDL-C significantly decreased post-treatment in Q1 and Q2 (P<0.05). Correlation analysis showed a positive correlation between ΔTSH and pre-treatment TSH levels (r=0.732, P<0.001). The proportion of patients with ≥70% TSH inhibition increased with higher pre-treatment TSH levels (P for trend <0.05). ΔLDL-C and ΔTSH were positively correlated (r=0.278, P<0.05), with ΔTSH identified as an influencing factor in multiple linear regression (β=0.133, 95% CI [0.042, 0.223], P<0.05).

**Conclusion:** Roxadustat effectively improves anemia in ESKD patients while inhibiting TSH and FT4 secretion and reducing TC and LDL-C levels. Decreases in TSH levels correlate with baseline TSH levels, and lowered blood lipid levels are associated with decreased TSH levels.

## Introduction

Anemia is a prevalent clinical manifestation in end-stage kidney disease (ESKD) and significantly affecting both the quality of life and disease progression^1^-^3^. Treatment for anemia is recommended by clinical practice guidelines^4^. Roxadustat, among the drugs used to treat renal anemia, has gained attention due to its regulatory effects on erythropoiesis and iron metabolism^5^-^8^. However, recent investigations suggest that roxadustat may influence thyroid hormone levels and blood lipid, necessitating a thorough exploration of its potential effects.

In a 2019 study, it was revealed that roxadustat shares a molecular structure resembling that of the thyroid hormone T3. It can bind to the thyroid hormone receptor β (TRβ) with a binding affinity greater than that of T3^9^. Subsequent case reports in 2021 indicated reversible serum low thyroid-stimulating hormone (TSH) hypothyroidism in ESKD patients following roxadustat treatment^10^-^12^. This effect may be attributed to roxadustat acting as a TRβ agonist, binding to pituitary TRβ, inhibiting TSH secretion, and decreasing free triiodothyronine (FT3) and free thyroxine (FT4) levels. A recent small retrospective clinical study demonstrated significant decreases in serum TSH, FT3, and FT4 levels after roxadustat treatment in patients with chronic kidney disease (CKD), undergoing hemodialysis, peritoneal dialysis, and those not on dialysis^13^-^15^. Cheng et al. reported over 50% TSH inhibition in 49% of patients, while the remaining 51% exhibited lower than 50% TSH inhibition 13. Clinical practice also showed differences in thyroid hormone inhibition respond to roxadustat in CKD patients, which may be influenced by various factors.

Patients with ESKD frequently experience euthyroid sickness syndromes, including low T3 and low T3/T4, impacting their quality of life and prognosis^16^, ^17^. Therefore, further analysis of roxadustat's effects on thyroid hormones is crucial. Additional research is warranted to assess whether roxadustat treatment affects the quality of life in ESKD patients.

Two phase 3 clinical studies in China showed that in addition to alleviating anemia, roxadustat significantly reduced total cholesterol (TC) and low-density lipoprotein cholesterol (LDL-C) in dialysis and CKD patients^18^, ^19^. While it is reasonable to speculate that this TC and LDL-C lowering effect of roxadustat may be attributed to the activation of the hypoxia-inducible factor (HIF) pathway^20^, comprehensive investigations on whether roxadustat's effects on blood lipid are linked to its T3 analog effects and the correlation between thyroid hormone and blood lipid levels are still lacking.

Therefore, our study explored the impact of roxadustat on thyroid hormones and blood lipid in ESKD patients undergoing hemodialysis, examining potential risk factors associated with these effects. Through this comprehensive analysis, we aim to contribute to a deeper understanding of the use of roxadustat in ESKD patients.

## Participants and Methods

### Study participants

Patients diagnosed with ESKD who underwent roxadustat treatment at the hemodialysis unit of Nanjing Drum Tower Hospital were recruited between September 2019 and September 2023. Inclusion criteria were as follows: individuals with ESKD, undergoing maintenance hemodialysis for a minimum of 2 months, experiencing concomitant renal anemia, and receiving roxadustat treatment for at least 2 months. Initially, a total of 95 patients meeting the enrollment criteria were selected. Seven with incomplete clinical data, four with a history of thyroid disease, one with malignant tumor progression, one with acute infection, one with newly onset acute cerebrocardiovascular disease, and one using medications affecting thyroid function were subsequently excluded. Ultimately, 80 patients were included in the study.

Among the participants, there were 55 males and 25 females, with ages ranging from 24 to 88 years and a mean age of (61.83±15.66) years. The duration of dialysis ranged from 2 to 105 months, with a median dialysis duration of 15.5 months. The roxadustat treatment period varied from 2 to 34 months, with a median duration of 9 months. Among the 80 patients, 58 opted for roxadustat treatment as their primary choice, while 22 switched to roxadustat due to side effector unsatisfactory correction of anemia after erythropoietin treatment. Throughout the observation period, 44 patients used statins, 25 used oral iron preparations, and 19 used sevelamer, with consistent dosages maintained.

This study was approved by the ethics committee of Nanjing Drum Tower Hospital and conformed to the ethics guidelines of the Declaration of Helsinki. All the participants signed the informed consent documentation.

### Study methods

#### Drug administration protocol for roxadustat

The consistent treatment regimen was used for the administration of roxadustat, whether initiated as the primary treatment or when patients transitioned from EPO to roxadustat. The initial roxadustat dose [Evrenzo, FibroGen (China) Medical Technology Development Co. Ltd] was orally administered at 100 mg (in patients weighing 45 to <60 kg) or 120 mg (in patients weighing ≥60 kg) three times weekly. Hemoglobin levels were monitored biweekly, and dosages were adjusted based on the hemoglobin level to sustain a hemoglobin concentration of 100-130 g/L. Once hemoglobin levels stabilized, patients underwent retesting every 4 weeks.

#### Questionnaire survey after roxadustat treatment

Evaluation of hypothyroidism-related symptoms post-roxadustat treatment was conducted to all patients. Questionnaire included decreased appetite, weight gain, fatigue and drowsiness, taciturn behavior, dull responses, cold extremities, constipation, dry skin, hair loss, increased menstrual volume, and hoarseness. A classification scale was utilized for scoring, with the magnitude of change for each symptom ranging from very much improved (+3), much improved (+2), slightly improved (+1), no change (0), slightly worse (-1), much worse (-2), to very much worse (-3). Patients selected the descriptor that best represented their symptom changes after treatment. Scores were summed to derive the overall score reflecting changes in hypothyroidism-related symptoms for each patient.

#### Anthropometric and biochemical measurements

Anthropometric parameters of height, weight were measured using standard equipment and techniques. Blood samples were obtained in the morning after a 12-hour overnight fast, both pre-treatment and post-treatment, and analyzed for hemoglobin (Hb), serum ferritin, serum iron, total iron binding capacity, albumin (ALB), triglycerides (TG), TC, high-density lipoprotein cholesterol (HDL-C), LDL-C, serum TSH, FT3, and FT4 levels.

A chemiluminescence microbead immunoassay (AU5421, Beckman Coulter, USA) was used to measure the serum ferritin concentration, serum iron concentration, and total iron binding capacity. A chemiluminescence immunoassay (ADVIA Centaure, Siemens, USA) was used to measure serum TSH, FT3, and FT4 levels.

### Statistical methods

SPSS 26.0 and GraphPad 8 were used for data processing and statistical analyses. Normally distributed quantitative data are expressed as the mean ± standard deviation, while non-normally distributed quantitative data are expressed as the median and quartile. Qualitative data are expressed as the frequency (percentage). Paired sample t tests or Wilcoxon rank sum tests were used for comparisons before and after treatment. One-way ANOVA was used for multiple group comparisons. Pearson and Spearman correlation analyses were used to assess correlation. Trend testing was performed by the Cochrane-Armitage trend test. Multiple linear regression analysis was conducted on markers that demonstrated significant differences. P value <0.05 was considered to indicate significance.

## Results

### Changes in Hb, blood lipid, and thyroid hormone levels before and after roxadustat treatment

After roxadustat treatment, the mean Hb concentration significantly increased from 84.96±16.20 to 102.43±19.38g/L (P<0.001). The serum ferritin concentration also significantly decreased from 182.2 (80.85, 286.73) to 102.80 (40.90, 168.73) ng/ml (P<0.001). Notably, there was no significant difference in transferrin saturation (TSAT)levels before and after treatment (P>0.05).

The TC level significantly decreased from 3.69±1.04 to 3.04±1.08 mmol/L(P<0.001), with a mean decrease of 0.71±1.04mmol/L. Additionally, LDL-C levels decreased significantly from 2.10±0.78 to 1.53±0.68mmol/L (P<0.001), with a mean decrease of 0.57±0.76mmol/L. However, there was no significant difference in TG and HDL-C levels before or after treatment (P>0.05).

Before roxadustat treatment, the TSH level of 64 patients (80%) fell within the normal range (0.27-4.2mIU/L), while 16 patients (20%) exhibited elevated TSH levels (>4.2mIU/L). Furthermore, 42 patients (52.5%) had FT3 levels below the normal range, and 16 patients (20%) had both FT3 and FT4 levels below the normal range. Post-roxadustat treatment, the TSH level of 17 patients (21.25%) decreased to less than 0.27mIU/L, while 14 patients (17.5%) did not experience a decrease. Additionally, 49 patients (61.25%) exhibited decreased FT3 levels below the normal range, along with 35 patients (43.75%) having both FT3 and FT4 levels below the normal range. Notably, there was no significant difference in the proportion of patients with low T3 levels before and after roxadustat treatment (P>0.05). However, the proportion of patients with low FT3/FT4 levels after treatment was significantly greater than that before treatment (P<0.001).

TSH levels significantly decreased from 2.01(1.26, 3.52) mIU/L before roxadustat treatment to 1.08(0.29, 1.88) mIU/L after treatment (P<0.001). FT4 levels decreased from 13.60±2.65pmol/L before treatment to 11.95±3.85pmol/L after treatment (P<0.001). While FT3 levels decreased from 3.02±0.67pmol/L before treatment to 2.88±0.78pmol/L after treatment, the difference was not statistically significant (P>0.05). There was no significant difference in symptom changes before and after treatment among the three groups categorized by post-treatment TSH suppression percentages, which were 2 (-4.00, 4.00), -2 (-5.00, 7.00), and -0.50 (-3.00, 3.00) respectively (Table 1, Table 2).

### Effects of TSH changes after roxadustat treatment on Hb, blood lipid, T3 and T4

To delve into the impact of TSH changes following roxadustat treatment on blood lipid, we categorized patients into tertiles based on post-treatment TSH suppression percentage [Q1 (≥70%), Q2 (30%-70%), and Q3 (≤30%)]. Comprehensive analysis revealed no significant differences in age, sex, weight, dialysis duration, roxadustat treatment duration or dose, or the proportion of patients using statins among the three groups (P>0.05).

Pre-treatment TSH levels in the Q1, Q2, and Q3 groups showed a gradual decrease, and these differences were statistically significant (P<0.05). However, there were no significant differences in pre-treatment levels of Hb, TG, HDL-C, TC, LDL-C, FT3, or FT4 among the three groups (P>0.05). After treatment, the TC, LDL-C, TSH, FT3, and FT4 levels in the three groups showed a gradual increase with decreasing TSH suppression, and these differences were significant (P<0.05). However, there were no significant differences in post-treatment TG or HDL-C levels among the three groups (P>0.05). The Hb levels in all groups were significantly higher after treatment (P<0.05), and there were no significant differences in post-treatment Hb levels among the three groups (P>0.05).

**Figure 1:**
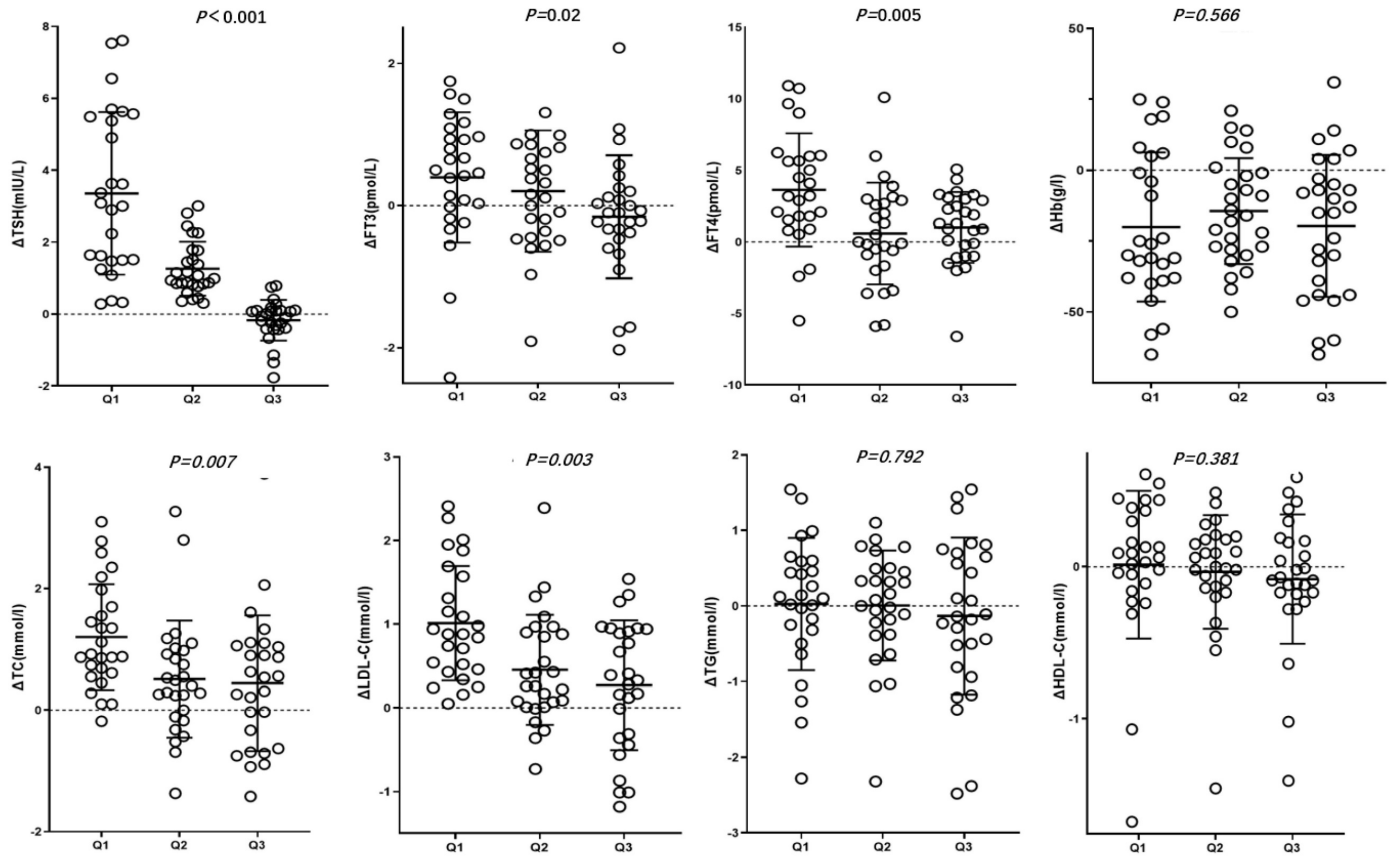
Changes in characteristics before and after roxadustat treatment in three groups based on post-treatment TSH suppression percentage Q1, Q2, and Q3. Δvalue was the difference of pre-treatment variables subtracting post-treatment ones.

In the Q2 and Q3 groups, post-treatment FT3 levels did not significantly differ from those before treatment (P>0.05). In the Q1 and Q2 groups, TSH, TC and LDL-C levels were significantly lower after treatment (P<0.05), whereas in the Q3 group, post-treatment TSH, TC and LDL-C levels did not significantly decrease (P>0.05). ΔTSH, ΔFT3, ΔFT4, ΔTC, ΔLDL-C, which is the changes in characteristics before and after roxadustat treatment were gradual decrease in Q1, Q2 and Q3 groups (P<0.05) (Table 2, Figure 1).

### 3. Correlations between changes in TSH before and after treatment and baseline data

We investigated the correlations between changes in TSH before and after treatment (ΔTSH) and baseline parameters, as well as the percentage of TSH suppression. ΔTSH showed a significant positive correlation with pre-treatment TSH (r=0.732, P<0.001), while it showed no significant correlation with age, sex, weight, dialysis duration, roxadustat treatment duration and dose, or pre-treatment FT3 and FT4 concentrations (all P>0.05). Similarly, the TSH suppression percentage was significantly positively correlated with pre-treatment TSH concentration (r=0.362, P<0.05), but not significantly correlated with other baseline parameters (P>0.05) (Table 3).

Patients were further stratified into tertiles (Q1, Q2, and Q3) based on pre-treatment TSH levels. The proportion of patients with a post-TSH change percentage ≥70% increased with rising pre-treatment TSH levels (P for trend <0.05). Age, sex, weight, dialysis duration, roxadustat treatment duration and dose did not significantly vary across tertiles (all P for trend >0.05) (Table 4).

### Correlation analysis of changes in LDL before and after roxadustat treatment (ΔLDL)

Correlation analysis revealed that ΔLDL-C before and after treatment was significantly positively correlated with ΔTC (r=0.860, P<0.001), as well as ΔTSH (r=0.278, P<0.05). However, no significant correlation was observed with ΔFT4 or ΔFT3 (P>0.05). ΔLDL-C did not significantly correlate with age, sex, weight, hemodialysis duration, roxadustat treatment duration, roxadustat dose, ΔHb, or the use of statins (P>0.05) (Table 5).

Multiple linear regression analysis was performed to analyze the influencing factors of LDL-C changes caused by roxadustat treatment. ΔTSH was found to be an influencing factor (β=0.133, 95% CI [0.042, 0.223], p<0.05). Age, ΔFT4, ΔFT3, ΔHb, roxadustat treatment duration and dose, and statin use did not significantly correlate with ΔLDL (all P>0.05). These findings show that LDL-C changes resulting from roxadustat treatment were positively correlated with changes in TSH levels.

## Discussion

Our findings indicate that roxadustat, a hypoxia-inducible factor-proline hydroxylase inhibitor (HIF-PHI), effectively treats renal anemia and significantly affects thyroid hormones (TSH and FT4) and lipid metabolism (TC and LDL-C levels). Roxadustat treatment led to increased hemoglobin levels and decreased serum ferritin, aligning with its known efficacy in addressing anemia by enhancing erythropoiesis and iron metabolism.

The study revealed a significant suppression of TSH and FT4 levels following roxadustat treatment, with a notable reduction in TC and LDL-C levels, while TG and HDL-C levels remained unchanged. Importantly, the extent of thyroid hormone suppression was positively correlated with baseline TSH levels, and a significant association was found between the decrease in LDL-C levels and the reduction in TSH levels.

Roxadustat's multifaceted mechanism of action, encompassing inhibition of HIF-PH, elevation of erythropoietin (EPO) concentrations, and enhancement of EPO receptor sensitivity, contributes to its efficacy in correcting anemia^7^, ^21^, ^22^. The study, conducted on anemic patients undergoing regular hemodialysis, showed a significant increase in hemoglobin levels and a reduction in serum ferritin levels after roxadustat treatment.

As roxadustat gains clinical prevalence, its impact on thyroid hormone levels, TC, and LDL-C levels has garnered attention. Our study supports the observation that roxadustat treatment significantly inhibits serum TSH and FT4 levels. Additionally, TC and LDL-C levels experienced a marked decrease, while TG and HDL-C levels showed no significant changes^5^, ^13^, ^18^. However, TSH inhibition was not observed in 17.5% of patients. Similarly, Cheng et al.'s study reported over 50% TSH suppression in approximately 49% of patients after roxadustat treatment^13^. This discrepancy highlights potential variations in treatment responses among different patient populations.

To investigate factors associated with TSH suppression, we categorized post-treatment TSH suppression percentages into tertiles. No significant differences in demographic or clinical parameters were observed among the groups, but a gradual decrease in serum TSH levels was noted. Significant inhibition of FT3 and FT4 levels was observed in the group with the highest TSH suppression. Correlation analyses revealed a significant positive correlation between changes in TSH levels before and after treatment and baseline TSH levels. Patients with higher baseline TSH levels were more likely to experience significant TSH suppression after roxadustat treatment.

While the presence of euthyroid sickness syndrome in CKD can influence patients' well-being and prognosis, the necessity of thyroxine replacement therapy remains debated^23^, ^24^. Some studies suggest that thyroxine replacement therapy may delay CKD progression and enhance quality of life and mental health in CKD patients with low T3/T4 syndrome^24^.Whether roxadustat-induced thyroid hormone suppression affects anemia improvement and results in hypothyroidism symptoms remains uncertain.

Our questionnaire results revealed that patients did not have hypothyroidism-related manifestations after roxadustat treatment, even in the presence of low T3 and low T4. Significant improvements in Hb levels were observed even in cases that TSH and FT3/FT4 were significantly suppressed after roxadustat treatment. These findings indicate that despite substantial thyroid hormone suppression, anemia improvement remained unaffected, and patients did not exhibit hypothyroidism symptoms, possibly due to the T3-like effects of roxadustat.

Our results are consistent with previous research indicating that roxadustat treatment can lead to reductions in TC and LDL-C levels^18^. Studies suggest that roxadustat activates the HIF pathway to induce degradation of 3-hydroxy-3-methylglutaryl-CoA reductase (HMGCR), thereby decreasing acetyl-CoA synthesis and ceramide levels^25^, ^26^, the drug has also been found to potentiate the effects of statins^27^. Additionally, the T3-like structure of Roxadustat may contribute to its lipid-lowering mechanisms. By regulating low-density lipoprotein gene expression via thyroid hormone response element (TRE), increases low-density lipoprotein clearance^28^. TRβ is highly expressed in the liver, and at least 90% of TRs are TRβ^29^. Notably, the high expression of TRβ in the liver allows roxadustat, with its T3-like structure, to bind to TRβ and exert lipid-lowering effects.

Our study also showed a significant decrease in TC and LDL-C levels after roxadustat treatment, while TG and HDL-C levels remained unchanged. This aligns with previous research indicating that roxadustat activates the HIF pathway to induce the degradation of HMG-CoA reductase, decreasing acetyl-CoA synthesis and ceramide levels. Roxadustat's T3-like structure may also contribute to its lipid-lowering mechanisms by regulating low-density lipoprotein gene expression and increasing low-density lipoprotein clearance.

The study analyzed lipid changes in groups with varying degrees of TSH suppression, revealing a likely correlation between TSH suppression and alterations in TC and LDL-C levels. Furthermore, we conducted correlation and multiple linear regression analyses to further examine the influencing factors of LDL-C decline. The findings indicate that changes in LDL-C levels were significantly influenced by changes in TSH levels, independent of other factors. This aligns with previous studies linking serum TSH concentration with blood lipid levels, particularly in hypothyroidism, where elevated TSH is often associated with high TC and LDL-C levels^18^, ^19^. This suggests that roxadustat's lipid-lowering effects may be mediated through thyroid hormone pathways.

In summary, while roxadustat effectively treats renal anemia, it also influences thyroid hormone and lipid metabolism. The observed correlations between TSH suppression and lipid changes highlight the need for further research to understand the broader implications of roxadustat treatment, especially its potential cardiovascular benefits and effects on low-T3 syndrome in CKD patients. Further extensive investigations are warranted to elucidate roxadustat's potential independent effects on cardiovascular health while rectifying anemia and its ability to ameliorate low-T3 syndrome in CKD through its T3 analog effects.

## Author contributions

Nan Li and Yun Hu designed the study. Nan Li and Wenxia Cui conducted the study, performed the analysis, wrote the manuscript and statistical analysis. Dinghuang Mu, Xiaoting Shi, Lei Gao and Sixiu Liu were involved in data collection and statistical analysis. Hengjin Wang and Chunming Jiang provided resources and supervised the study. Yun Hu supervised the study and critically reviewed the manuscript for important intellectual content.

## Figures and Tables

**Table 1:** Changes in characteristics before and after roxadustat treatment

Characteristic	Pre-treatment	Post-treatment	*P*
Age, (year)	61.83±15.66	62.02±15.43	0.912
Male, n (%)	55(68.75%)	55(68.75%)	1
Weight, (kg)	65.80±12.38	65.35±13.45	0.895
Hemodialysis duration, (Month)	15.550(5.00, 34.00)	17.00(5.00, 34.00)	0.324
Treatment Time, (Month)	NA	9.00(3.25, 15.75)	NA
Dose, (mg/W)	300(210, 300)	210(150, 270)	0.023
Hemoglobin, (g/l)	84.96±16.20	102.43±19.38	<0.001
Ferritin, (ng/ml)	182.20(80.85, 286.73)	102.80(40.9, 168.73)	<0.001
TSAT, (%)	27.33±14.11	24.00±12.04	0.151
Triglyceride, (mmol/L)	1.37±0.64	1.41±0.92	0.758
Total cholesterol, (mmol/L)	3.69±1.04	3.04±1.08	<0.001
HDL-C, (mmol/L)	0.95±0.36	0.98±0.47	0.698
LDL-C, (mmol/L)	2.10±0.78	1.53±0.68	<0.001
TSH, (mIU/L)	2.01(1.26, 3.52)	1.08(0.29, 1.88)	<0.001
FT3, (pmol/L)	3.02±0.67	2.88±0.78	0.086
FT4, (pmol/L)	13.60±2.65	11.95±3.85	<0.001
Medicine			
Statins, n (%)	44(55)	44(55)	1
ACEI/ARB, n (%)	29(36.25)	29(36.25)	1
Oral iron, n (%)	25(31.25)	25(31.25)	1
Sevelamer, n (%)	19(23.75)	19(23.75)	1

**Table 2:** Changes in characteristics before and after roxadustat treatment in three groups based on post-treatment TSH suppression percentage Q1, Q2, and Q3

	Q1(≥70%) (N=27)	Q2(30%-70%) (N=27)	Q3(≤30%) (N=26)	*P*
Age(y)	64.19±20.00	62.74±11.13	58.42±14.53	0.327
Male, n (%)	19(70.37%)	17(62.96%)	20(76.92%)	0.680
Weight(kg)	67.46±10.32	65.36±13.23	64.54±13.65	0.492
Hemodialysis duration(M)	13.00(4.00,23.00)	15.00(4.00,38.00)	21(5.00,45.25)	0.495
Treatment duration(M)	8.50(3.00,14.00)	10.00(4.00,18.00)	9.50(3.00,17.00)	0.822
Dose(mg/W)	261.48±65.50	251.11±55.91	257.31±59.50	0.690
Statins, n (%)	13(48.15%)	16(59.25%)	139(59.00%)	0.682
**Hb**				
Pre-treated	79.59±14.73	87.81±16.97	86.50±16.93	0.142
Post-treated	99.78±18.79	101.93±19.12	104.14±21.75	0.530
*P*	0.001	<0.001	0.001	
**TC**				
Pre-treated	3.67±0.96	3.67±1.02	3.76±1.03	0.924
Post-treated	2.40±0.60	3.28±1.16	3.30±1.04	<0.001
*P*	<0.001	0.015	0.060	
**LDL-C**				
Pre-treated	2.21±0.80	2.04±0.77	2.05±0.78	0.851
Post-treated	1.15±0.35	1.65±0.76	1.80±0.68	0.001
*P*	<0.001	0.001	0.144	
**TG**				
Pre-treated	1.39±0.64	1.34±0.53	1.39±0.76	0.874
Post-treated	1.34±0.97	1.45±0.92	1.43±0.91	0.663
*P*	0.435	0.99	0.909	
**HDL-C**				
Pre-treated	0.85±0.33	0.99±0.34	1.00±0.39	0.210
Post-treated	0.83±0.48	1.04±0.46	1.08±0.68	0.058
*P*	0.163	0.949	0.509	
**TSH**				
Pre-treated	3.38(1.68, 5.69)	2.39(1.35, 3.78)	1.60(1.07, 2.10)	0.004
Post-treated	0.12(0.04, 0.37)	1.46(0.66, 2.08)	1.81(1.39, 2.49)	<0.001
*P*	<0.001	<0.001	0.118	
**FT3**				
Pre-treated	2.96±0.64	23.07±0.63	3.03±0.75	0.953
Post-treated	2.55±0.83	2.92±0.63	3.18±0.75	0.006
*P*	0.009	0.341	0.326	
**FT4**				
Pre-treated	13.58±2.75	13.33±3.03	13.86±2.11	0.792
Post-treated	9.71±3.87	13.03±3.14	12.90±2.69	0.001
*P*	<0.001	0.559	0.040	
**Changes in symptoms**	2(-4.00, 4.00)	-2(-5.00, 7.00)	-0.50(-3.00, 3.00)	0.682

**Table 3:** Correlations between ΔTSH before and after treatment and baseline data

	ΔTSH		Post-treatment TSH suppression percentage	
	r	*P*	r	*P*
Age	0.214	0.057	0.191	0.089
Sex	0.108	0.34	0.027	0.809
Weight	0.011	0.926	0.051	0.656
Hemodialysis duration	-0.11	0.332	-0.116	0.307
Treatment duration	0.005	0.963	0.05	0.66
Dose(mg/W)	0.019	0.864	0.014	0.902
Alb	-0.016	0.157	-0.193	0.086
Hb	-0.266	0.017	-0.152	0.178
TSH	0.732	<0.001	0.362	0.001
FT3	0.030	0.791	0.023	0.840
FT4	-0.068	0.548	-0.105	0.353

**Table 4:** Baseline characteristics in three groups based on pre-treatment TSH levels Q1, Q2, and Q3

	Q1(≤1.570) (N=27)	Q2(1.571-2.840) (N=27)	Q3(≥2.841) (N=26)	Trend *P*
Age (y)	59.30±13.95	61.30±15.06	64.46±17.00	0.286
Male, n (%)	21(77.78%)	19(70.37%)	15(57.69%)	0.326
Weight (kg)	66.82±13.21	65.69±11.95	64.87±12.32	0.512
Hemodialysis duration (M)	20.00(5.00, 48.00)	21.00(8.00, 31.00)	8.5(4.00, 35.50)	0.403
Treatment duration (M)	5.00(3.00, 11.00)	9.00(2.00, 17.00)	7.50(3.50, 14.00)	0.503
Dose (mg/W)	261.11±58.73	252.22±58.99	256.54±63.75	0.673
Hb (g/l)	84.81±17.81	90.22±16.24	78.58±13.31	0.191
TG (mmol/l)	1.47±0.69	1.39±0.61	1.26±0.63	0.102
TC (mmol/l)	3.52±1.02	3.70±1.01	3.88±0.95	0.110
HDL-C (mmol/l)	0.79±0.27	0.97±0.41	1.08±0.32	0.026
LDL-C (mmol/l)	1.99±0.74	2.10±0.39	2.22±0.82	0.184
TSH (mIU/L)	2.04(1.30, 3.52)	2.06(1.76, 2.49)	4.84(3.52, 5.82)	<0.001
FT3 (pmol/L)	2.91±0.71	3.11±0.60	3.05±0.70	0.151
FT4 (pmol/L)	13.40±3.05	13.99±2.43	13.37±2.44	0.094
Post-TSH change percentage<30% (n,%)	13(48.15)	12(44.44)	2(7.41)	0.002
Post-TSH change percentage 30%-70% (n, %)	8(29.63)	10(38.46)	9(34.61)	0.699
Post-TSH change percentage ≥70% (n, %)	6(23.07)	5(19.23)	15(57.69)	0.007

**Table 5:** Correlation analysis of changes in LDL before and after roxadustat treatment(ΔLDL)

	r	*P*
Age	0.008	0.941
Sex	0.042	0.711
Weight	-0.063	0.579
Hemodialysis duration	-0.028	0.804
Statins	-0.078	0.493
Treatment duration	-0.064	0.57
Dose	0.158	0.16
ΔTSH	0.278	0.013
ΔFT3	0.182	0.107
ΔFT4	0.134	0.236
ΔHb	-0.050	0.661
ΔTC	0.860	<0.001

## Abbreviations

ESKD: end-stage kidney disease
TRβ: thyroid hormone receptor beta
TSH: thyroid stimulating hormone
FT3: free triiodothyronine
FT4: free thyroxine
CKD: chronic kidney disease
TC: total cholesterol
LDL-C: low-density lipoprotein cholesterol
HIF: hypoxia-inducible factor
Hb: hemoglobin
ALB: albumin
TG: triglycerides
HDL-C: high-density lipoprotein cholesterol
TSAT: transferrin saturation
HIF-PHI: hypoxia inducible factor-proline hydroxylase inhibitor
EPO: erythropoietin
HMGCR: hydroxy-3-methylglutaryl-CoA reductase
TRE: thyroid hormone response element
